# FcgRIIb mediates amyloid-β neurotoxicity and memory impairment in a model of Alzheimer’s disease

**DOI:** 10.1186/1750-1326-8-S1-P22

**Published:** 2013-09-13

**Authors:** Yong-Keun Jung

**Affiliations:** 1 School of Biological Sciences, Seoul, Republic of Korea

## 

Amyloid-β (Aβ) induces neuronal loss and cognitive deficits and is believed to be a prominent cause of Alzheimer’s disease (AD). However, the cellular mechanism of the pathogenesis is not fully understood. Here we report that Fcg-receptor IIb (FcgRIIb) mediates Aβ neurotoxicity and neurodegeneration. We found that FcgRIIb is significantly up-regulated in the hippocampus of AD brains and neuronal cells exposed to Aβ_1-42_*.* Neuronal FcgRIIb activates ER stress and caspase-12, and FcgRIIb knockout primary neurons are resistant to Aβ_1-42_-induced cell death in vitro. FcgRIIb deficiency ameliorates Aβ_1-42_-induced inhibition of long-term potentiation and inhibits the reduction of synaptic density by naturally secreted Aβ. Moreover, genetic depletion of FcgRIIb rescues the memory impairments in AD model mice. In an action mode of FcgRIIb in Aβ neurotoxicity, we found that soluble Aβ_1-42_ oligomers interact with FcgRIIb in vitro and in AD brains, and inhibition of their interaction blocks Aβ_1-42_ neurotoxicity. Thus, we conclude that FcgRIIb has an aberrant but essential role in Aβ_1-42_-mediated neuronal dysfunction, providing insight into Aβ neuropathology.

